# Thermal Quenching of Intrinsic Photoluminescence in Amorphous and Monoclinic HfO_2_ Nanotubes

**DOI:** 10.3390/ma17225587

**Published:** 2024-11-15

**Authors:** Artem Shilov, Sergey Savchenko, Alexander Vokhmintsev, Kanat Zhusupov, Ilya Weinstein

**Affiliations:** 1 NANOTECH Centre, Ural Federal University, Mira Str., 19, 620002 Ekaterinburg, Russia; ao.shilov@urfu.ru (A.S.); s.s.savchenko@urfu.ru (S.S.); a.s.vokhmintsev@urfu.ru (A.V.); zhusupov.kanat@mail.ru (K.Z.); 2 Higher School of Metallurgy and Mining, Rudny Industrial University, 50 Let Oktyabrya Str., 38, Rudny 111500, Kazakhstan; 3 Institute of Metallurgy, Ural Branch of the RAS, Amundsena Str., 101, 620108 Ekaterinburg, Russia

**Keywords:** hafnia nanotubes, anion deficiency, electrochemical anodization, photoluminescence thermal quenching, thermally stimulated processes, luminescence intensity ratio, activation energy, non-stoichiometric hafnium dioxide, oxygen vacancy

## Abstract

Nanotubular hafnia arrays hold significant promise for advanced opto- and nanoelectronic applications. However, the known studies concern mostly the luminescent properties of doped HfO_2_-based nanostructures, while the optical properties of nominally pure hafnia with optically active centers of intrinsic origin are far from being sufficiently investigated. In this work, for the first time we have conducted research on the wide-range temperature effects in the photoluminescence processes of anion-defective hafnia nanotubes with an amorphous and monoclinic structure, synthesized by the electrochemical oxidation method. It is shown that the spectral parameters, such as the position of the maximum and half-width of the band, remain almost unchanged in the range of 7–296 K. The experimental data obtained for the photoluminescence temperature quenching are quantitatively analyzed under the assumption made for two independent channels of non-radiative relaxation of excitations with calculating the appropriate energies of activation barriers—9 and 39 meV for amorphous hafnia nanotubes, 15 and 141 meV for monoclinic ones. The similar temperature behavior of photoluminescence spectra indicates close values of short-range order parameters in the local atomic surrounding of the active emission centers in hafnium dioxide with amorphous and monoclinic structure. Anion vacancies $V_{O}^{-}$ and $V_{O}^{2-}$ appeared in the positions of three-coordinated oxygen and could be the main contributors to the spectral features of emission response and observed thermally stimulated processes. The recognized and clarified mechanisms occurring during thermal quenching of photoluminescence could be useful for the development of light-emitting devices and thermo-optical sensors with functional media based on oxygen-deficient hafnia nanotubes.

## 1. Introduction

Hafnia nanotubes have a unique combination of physico-chemical properties and, according to calculations [1,2], demonstrate high chemical and thermal stability. In addition, it has been experimentally shown that nanotubular morphology is maintained in the temperature range from room temperature to 900 °C [3]. Due to the high atomic mass of hafnium, its structures demonstrate inherent resistance to ionizing radiation [4]. Currently, the electrochemical oxidation method is broad-reaching to synthesize nanoporous and nanotubular matrices based on wide-gap oxides of transition metals (HfO_2_, ZrO_2_, TiO_2_, etc.). It is well-designed for effective scaling and creating appropriate, functional, and commercial-volume media for a wide variety of high applications [5,6,7,8,9]. Specifically, HfO_2_-based 1D-structures grown-up by anodization have outstanding optical, electronic, and electro-physical properties and can be utilized for manufacturing non-volatile memory devices and light-emitting matrices [10,11,12,13,14,15,16,17].

It is known that anion-deficiency hafnia nanostructures maintain their own photoluminescence (PL) in the blue region. In addition, the morphology, phase composition, and synthesis method dramatically affect their spectral characteristics [18,19,20,21]. HfO_2_ is an extremely efficient, stable matrix for incorporating rare earth ions because of its low optical absorption in the visible spectrum, the possibility of adjusting and manipulating luminescent response parameters, and the high atomic mass of hafnium. Hafnium dioxide with a monoclinic structure is the most stable modification, preserved in a wide temperature range: the monoclinic phase transforms into a tetragonal phase when heated above 1700 K and into a cubic phase at T > 2500 K [22]. The listed features attract the attention of researchers and provide new avenues for designing advanced fast scintillators [23,24,25,26] and thermo-optical sensors [27,28].

Despite active studies of the luminescent properties of HfO_2_-based nanostructures, most works concern doped matrices [25,29,30,31], while the optical properties of nominally pure hafnia with optically active centers of intrinsic nature are far from being sufficiently investigated. In addition, the usage of HfO_2_ as a solid base for light-emitting media, including in high-power LEDs [32], requires thoroughly understanding how the luminescent response behaves under conditions of local overheating/cooling or jumps in the operating temperature [33,34]. Previously, the papers have explored the temperature influence on the luminescent properties of undoped HfO_2_ as thin films [35] and nanopowders [36]. In these cases, the authors compared the spectra measured at two temperatures—room temperature and 10 K. The paper [37] analyzed the PL spectral parameters for HfO_2_ nanopowder upon excitation by photons with energies > 4.4 eV. Upon cooling < 150 K, the detected shift of the emission spectrum to the red region appears to be incidental with a change in the configuration of the luminescence center. It should be emphasized that research on PL temperature quenching processes in hafnia nanotubes has not been previously conducted; at least, we have not been able to find any analogous sources within a targeted literature look-through.

In our previous work [38], we studied the photoluminescence (PL) of hafnia nanotubes at room temperature and at 10 K. However, a comprehensive understanding of the thermal quenching processes requires detailed measurements across the entire range from cryogenic to room temperatures. Elucidating the mechanism of thermal PL quenching is crucial for the targeted synthesis of highly efficient blue luminophores based on nanotubular HfO_2_, as well as for the potential application of hafnia nanotubes in the development of luminescent temperature sensors. The present paper explores for the first time the features of photoluminescence of anion-deficient hafnia nanotubes with an amorphous and monoclinic crystalline structure over the temperature range of 7–296 K.

## 2. Materials and Methods

In this work we study the properties of as-grown and annealed hafnia nanotubes. The nanotubular samples were synthesized earlier [38] by using electrochemical anodization of the hafnium foil (HFI-1 grade, 99.9% purity, MetallKomplekt LLC, Moscow, Russia), where Hf acted as the anode and stainless steel was used as the cathode. The anodization was carried out in the solution of NH_4_F (0.5 wt%, 98.5% purity, Klassik LLC, Moscow, Russia), H_2_O (2 wt%), and ethylene glycol (99.5% purity, EKOS-1 JSC, Moscow, Russia) [39]. H_2_O acts as an oxygen source during the electric field-assisted oxidation at the metal/oxide interface, while NH_4_F provides F^−^ ions, dissolving the oxide to obtain nanotubes [5]. One can tailor the anodic oxide morphology by decreasing the electrolyte dielectric constant, to lower the oxidation rate, and to obtain nanotubular structure. The decrease of the electrolyte viscosity leads to increasing the length of the oxide layer since the ionic diffusion is improved [5]. We use ethylene glycole as the electrolyte since it leads to a faster oxidation process than a dissolution, thus forming a nanotubular structure. The synthesis was performed under a constant voltage of 40 V for 4 h. The synthesized samples contain carbon and fluorine as a part of the electrolyte used in their synthesis. These precursor residues can be removed after heating hafnia NT to 300 °C, resulting in obtaining an as-grown sample. Additionally, the other NT sample was annealed in air at 700 °C for 2 h.

Sample morphology was investigated using Carl Zeiss SIGMA VP (Oberkochen, Germany) scanning electron microscope (SEM) with an Oxford Instruments X-Max 80 (Abingdon, UK) module for energy dispersive analysis (EDS) and transmission electron microscope (TEM) JEOL JEM-2100 (Tokyo, Japan). To study the temperature effect on the PL spectra, the samples were placed into a Janis CCS 100/204 N (LakeShore, Westerville, OH, USA) closed cycle refrigerator coupled with a LakeShore DT-670B-CU temperature sensor (LakeShore, Westerville, OH, USA) and Model 335 controller (LakeShore, Westerville, OH, USA) [40]. Recently [38] we analyzed the photoluminescence (PL) spectra of synthesized samples at two temperatures—10 and 280 K. To study thermal quenching processes in this work, the PL of the hafnia NTs was measured within a 7–296 K temperature range (the spectra were recorded at 7 K, every 10 K in the range of 10–100 K, and every 20 K in the range of 100–296 K) using an Andor Shamrock SR-303i-B spectrograph (Belfast, UK) with a NewtonEM DU970P-BV-602 CCD (Andor Technologies, Belfast, UK) recording array [41]. To negate possible thermoluminescence contribution upon heating, the measurements were performed while cooling the samples. The input slit was 100 μm with a diffraction grating with a groove density of 150 L/mm. The exposure time was 0.1 s for monoclinic NT and 0.3 s for amorphous NT. A DTL-389QT laser (Laser-Compact Group, Moscow, Russia) with a wavelength of 263 nm (4.71 eV) was used as a photoexcitation source. To plot the obtained PL spectra against photon energy, a spectral correction was performed [42].

## 3. Results

**Figure 1:**
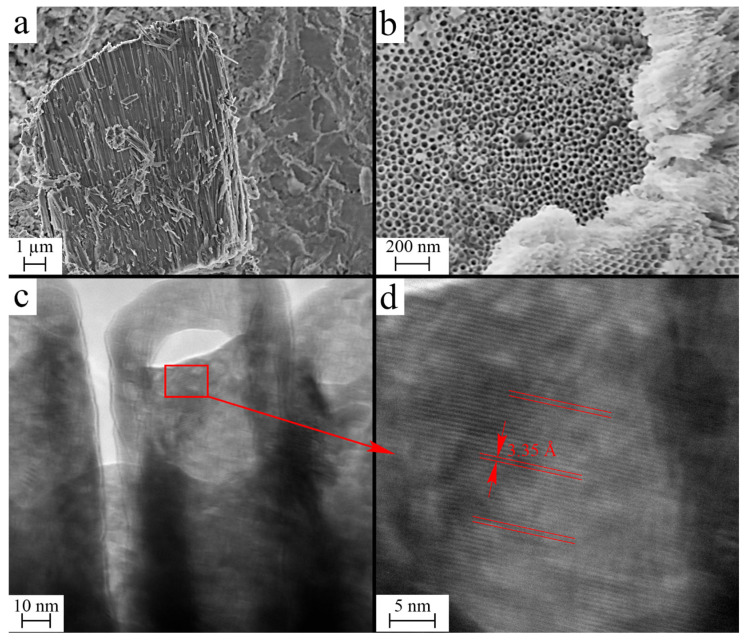
Scanning electron microscope (SEM) (**a**,**b**) and transmission electron microscope (TEM) (**c**,**d**) images obtained for the monoclinic HfO_2_ nanotubes under study. The value marked in (**d**) corresponds to the interplanar distance $\bar{1}11$.

Figure 1 displays images from scanning and transmission electron microscopes. The average length and diameter of the synthesized nanotubes amount to 10 ± 3 μm and 46 ± 7 nm, respectively (the size distribution histograms of wall thickness, and inner and outer diameters, are shown in Figure S1 in the Supplementary Materials). The EDS chemical analysis method reveals no impurities of heavy elements in the samples, and the O/Hf ratio falls within the 1.78–1.91 range, indicating anionic non-stoichiometry. The original samples include fluorine and carbon as the fragments of the precursors applied during synthesis of the nanotubes (NTs). Upon being heated up to 300 °C, they were effectively eliminated. The XRD and Raman spectroscopy data attest to the fact that the initial nanotubes are amorphous. After annealing for 2 h at 700 °C in an air atmosphere, the nanotubes crystallize to form the most stable monoclinic phase of hafnia (see Figure S2 in the Supplementary Materials) [38]. Figure 1d clearly shows the interplanar distance of 3.35 Å, corresponding to the planes with $\bar{1}11$ Miller indices in hafnia of a monoclinic crystal structure. Selected area electron diffraction and Fast-Fourier Transformed image (see Figures S3 and S4 in the Supplementary Materials) also confirm the crystalline nature of the annealed HfO_2_ nanotubes.

**Figure 2:**
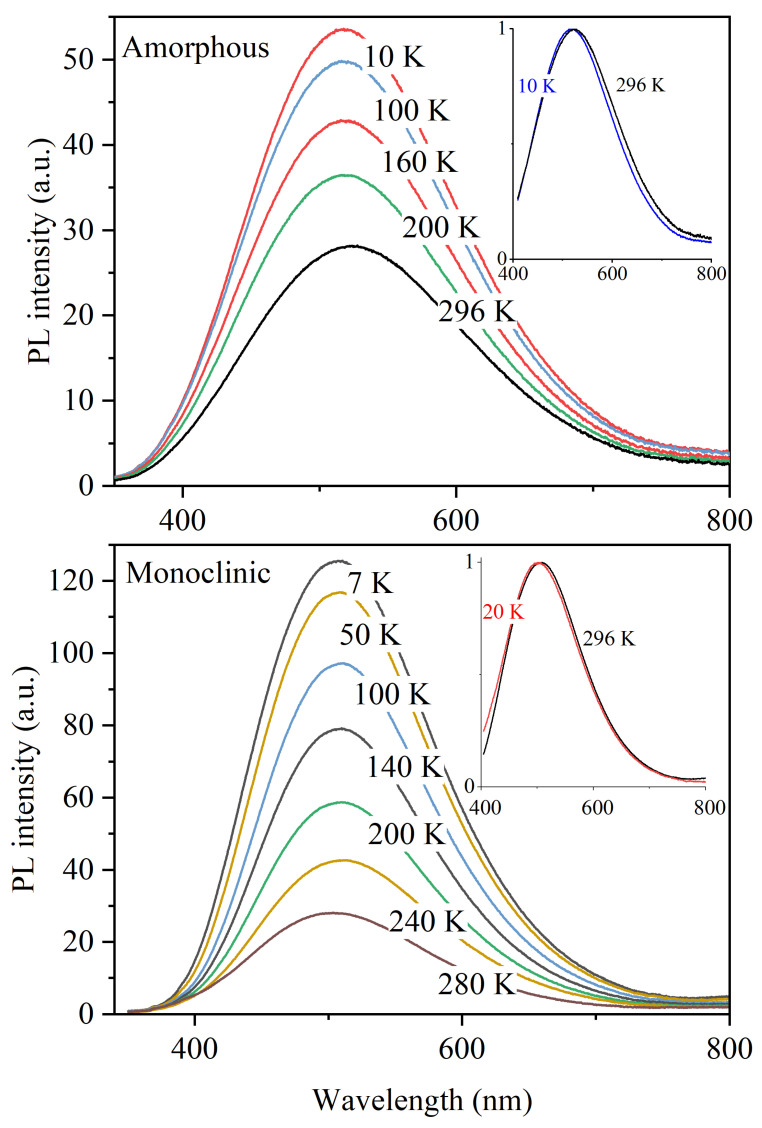
Photoluminescence (PL) spectra of amorphous (**top**) and monoclinic (**bottom**) hafnia nanotubes measured at different temperatures.

When excited by UV photons, nanotubular HfO_2_ exhibited emission in the blue–green region of the spectrum. Figure 2 shows the spectra for amorphous samples (top) and for the HfO_2_ nanotubes with a monoclinic structure (bottom), measured at different temperatures. The spectra for amorphous nanotubes are a broad band with a maximum in the region of E_max_ = 2.28 ± 0.02 eV and a half-width of FWHM = 0.82 ± 0.02 eV. In the case of monoclinic NTs, E_max_ = 2.37 ± 0.02 eV and FWHM = 0.73 ± 0.02 eV. It is seen that cooling provokes an increase in luminescence intensity; however, the position and shape of the spectral band are weakly temperature-dependent (see insets in Figure 2).

**Figure 3:**
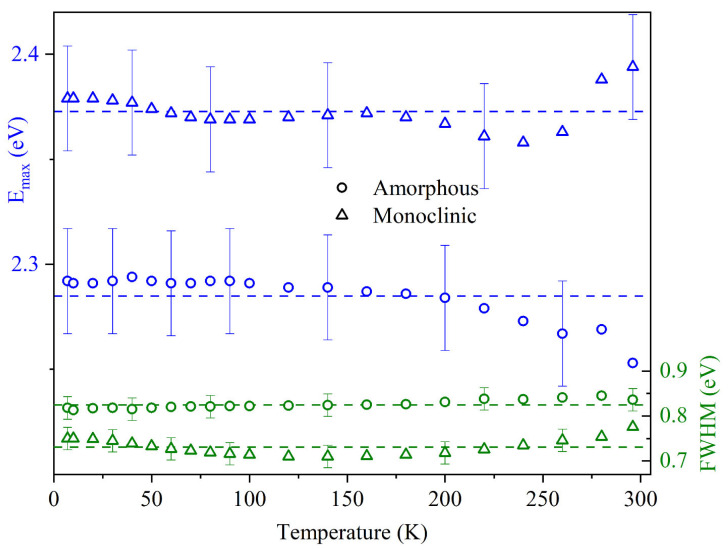
Temperature dependencies of the experimental values of the maximum position E_max_ (blue color) and half-width FWHM (green color) of the measured PL bands. The circles indicate data for amorphous NTs, triangles—for monoclinic NTs. The dashed lines show the averaged values of E_max_ and FWHM in the temperature range of 7–296 K.

At 20 K, the PL intensity reaches its maximum, and further cooling virtually leaves it unchanged. At 7–20 K, compared to room temperature, luminescence intensity doubles for amorphous NTs and is higher by a factor of 7 for monoclinic NTs. The synthesized samples’ PL spectral parameter values are shown against the temperature in Figure 3. As can be clearly seen, the position of the spectral band and its half-width for both amorphous or monoclinic NTs remain unchanged over the entire temperature range of 7–296 K with an inaccuracy of 0.02 eV. Furthermore, a noticeable shift of ≈0.1 eV toward the blue region is observed in the PL spectrum of monoclinic NTs.

## 4. Discussion

**Figure 4:**
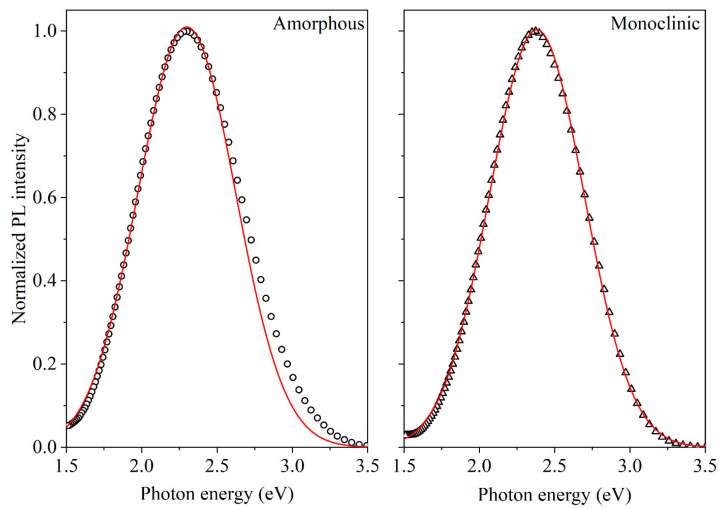
PL spectra of amorphous (**left**, circles) and monoclinic (**right**, triangles) nanotubes measured at a temperature of 10 K, with decomposition into Gaussian components (red lines).

All the measured PL spectra of nanotubular HfO_2_ can be described by a wide single Gaussian band. Figure 4 exemplifies an approximation of the registered spectra. It should be noted that, for monoclinic NTs, the values of the Gaussian’s maximum and half-width coincide with the experimental ones, whereas, for amorphous NTs, the PL band is not symmetrical and is characterized by a stretched section in the high-energy region. The parameters of the Gaussian curves (E_max_ and FWHM) responsible for the PL spectra of amorphous and monoclinic NTs turn out to be quite close to each other; for comparison, they are given in Table 1.

Based on anion vacancies in the samples, optically active centers cause the observable PL [38,43,44]. This is compatible with the oxygen deficiency revealed by the EDS method; see Section 3. The PL of other HfO_2_ morphological modifications (thin films, nanopowders, and nanocrystals) experiences a shift in the blue region of the spectrum regarding PL in the current paper. The observable shift can be associated with various synthesis methods and the different morphologies of the HfO_2_ nanostructures tested, as well as with different kinds and energies of excitation. So, a wide band of 2.6–2.7 eV appears in the PL spectra of 50 nm thin amorphous HfO_2_ films excited by photons of 5.2–5.4 eV [21]. For nanopowders with a monoclinic structure produced by solution combustion synthesis, upon excitation by photons with hν > 3.8 eV, luminescence arises near 2.8 eV [36]. With hafnia nanotubes prepared by radio frequency sputtering with electrospun polyvinylpyrolidone nanofibers as templates with a diameter of 200–250 nm and a wall thickness of 25–55 nm, the emission maximum is recorded in the region of 425 nm (2.91 eV) [45].

Meantime, for HfO_2_ nanopowders and nanocrystals fabricated by the sol–gel method, PL is observed in the region of 2.4–2.5 eV. Similar emission is inherent to a monoclinic nanopowder subjected to annealing at a temperature of 1000 °C and at excitation energies of ≥4.4 eV [37]. As reported in [46], under excitation by 325 nm (3.82 eV) laser radiation, the PL spectrum of nanopowders with a grain size in the range of 67–161 nm reveals two peaks near 2.4 eV and 3.0 eV.

According to calculations [47] and experimental diffuse reflection spectra [38,40], it is known that oxygen vacancies in hafnium dioxide absorb excitation radiation of hν > 3 eV. In the studies cited above, various photoexcitation energies > 3.8 eV are used to detect the PL of oxygen vacancies, which also aligns well with UV–Vis spectroscopy data and theoretical calculations. The previously measured PL excitation spectra in hafnium dioxide nanotubes [38] contain 4 maxima: 4.6, 4.8, 5.1 and 5.5 eV. The first three peaks correspond to the sub-band excitation of oxygen vacancies, and the latter at 5.5 eV is in the region of direct band-to-band transitions that form the intrinsic optical absorption edge [38]. In this work, excitation with photons of 4.71 eV energy is used, leading to electronic transitions from the valence band to the energy levels of oxygen vacancies located near the bottom of the conduction band [48].

It is worth pointing out that the PL observed in HfO_2_ nanotubes agrees quite well with the cathodoluminescence (CL) spectra measured previously at room temperature [38]. The CL spectrum of amorphous nanotubes is a broad band at 2.40 ± 0.02 eV with a half-width of 1.32 ± 0.02 eV. Further high-temperature annealing provokes a narrowing of the spectrum FWHM = 0.93 ± 0.02 eV and its slight blue shift to 2.45 ± 0.02 eV. A similar emission of 2.50 eV was observed in [49] during a study of the radioluminescence (RL) of monoclinic HfO_2_ nanocrystals with an average diameter of 2.8 nm obtained by nonaqueous sol–gel synthesis and annealed at 700 °C. For ease of comparison, all the data are collected in Table 1.

**Table 1:** Spectral parameters of the luminescence of hafnia nanostructures.

Morphology, Synthesis Method, Characteristic Size, nm	Luminescence Type	λ_exc_, nm	Temperature, K	Spectral Parameters		Reference
				E_max_, eV	FHWM, eV	
Amorphous nanotubes, anodic oxidation, outside diameter = 46 ± 7	PL	263	7–296	2.30 ± 0.02	0.76 ± 0.02	This work
Monoclinic nanotubes, anodic oxidation, outside diameter = 46 ± 7	PL	263	7–296	2.38 ± 0.02	0.74 ± 0.02	This work
Monoclinic nanopowder, sol–gel, diameter = 67 ± 15	PL	275	296	2.60 ± 0.02	0.58 ± 0.02	[40]
Monoclinic nanopowder, sol–gel, crystallite size = 29 ± 3	PL	280	10–300	2.33–2.48 ***	0.51–0.65 ***	[37]
Monoclinic nanopowder, sol–gel, grain size = 67–161	PL	325	296	2.4 3.0	–	[46]
Amorphous nanotubes, anodic oxidation, outside diameter = 46 ± 7	CL *	—	296	2.40 ± 0.02	1.32 ± 0.02	[38]
Monoclinic nanotubes, anodic oxidation, outside diameter = 46 ± 7	CL *	—	296	2.45 ± 0.02	0.93 ± 0.02	[38]
Monoclinic nanocrystals, sol–gel, diameter = 2.8	RL **	—	10–300	2.14 ± 0.01 2.50 ± 0.01 2.94 ± 0.03	0.75 ± 0.02 0.67 ± 0.03 0.61 ± 0.02	[49]

* Cathodoluminescence, accelerating voltage 20 kV. ** Radioluminescence, X-rays, 20 kV. *** Our estimates based on the data given in the cited paper.

Typically, as the temperature increases, the spectral parameters of optical bands in materials with a dominant ionic bond type (the ionicity of the bond in HfO_2_ is 68.17% [50]) undergo characteristic changes. Due to strong electron–phonon interaction, the peak maximum experiences red-shift and its FWHM increases [40,51]. These changes are a characteristic behavior when the phonon-induced displacement of energy levels occurs. In our case, as shown in Figure 2, these parameters remain unchanged across the entire temperature range from 7 to 296 K. This behavior may be attributed to mechanisms of charge redistribution between traps during excitation relaxation, as well as possible interactions of electrons and holes with the energetic or vibrational states of trapping and recombination centers in the radiative transition processes.

**Figure 5:**
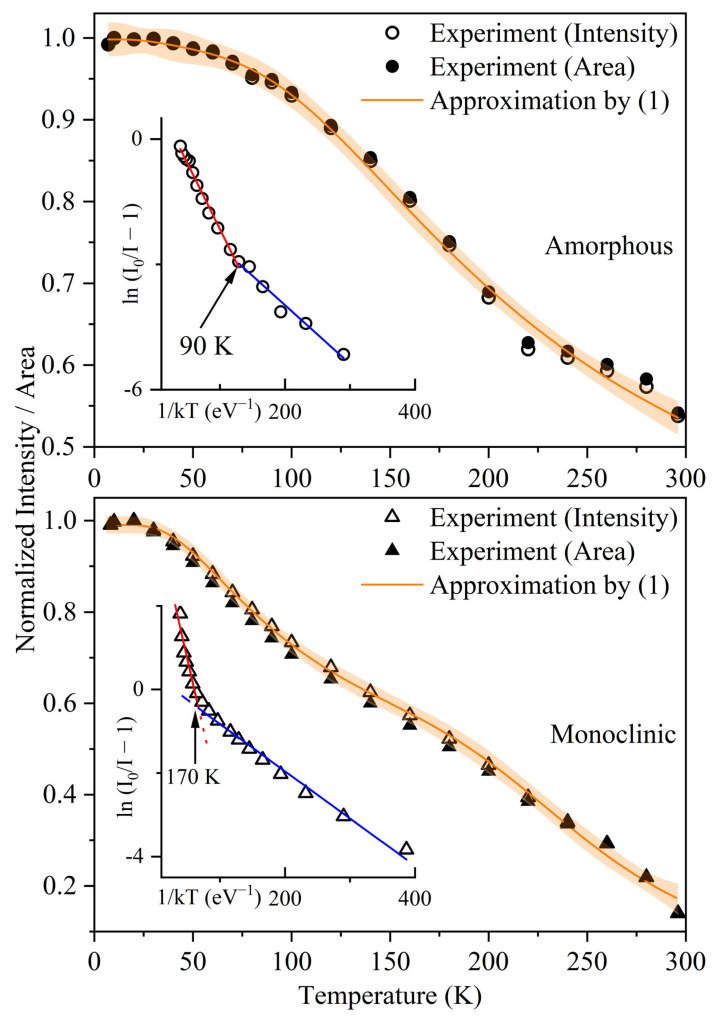
Dependence *I*(*T*) for amorphous (**top**) and monoclinic (**bottom**) NTs. The red and blue lines, see insets, are linear approximations.

To get a quantitative estimate of the temperature influence on the PL processes in the samples tested, all the measured spectra were normalized to the highest intensity *I* at 20 K. The temperature dependencies *I*(*T*) built in Arrhenius coordinates contain two linear sections that appear to be caused by two thermal activation barriers (see insets in Figure 5). Thus, with two separate channels of nonradiative relaxation of excitation, the experimental data *I*(*T*) were described within the Mott relation for temperature quenching of photoluminescence [52,53,54]:

(1) $$I(T)=\frac{I_{0}}{1+S_{1}\exp(-\frac{E_{q1}}{kT})+S_{2}\exp(-\frac{E_{q2}}{kT})}$$

where *I*_0_ is the PL intensity at 0 K; *S*_1_ and *S*_2_ are dimensionless pre-exponential factors; *E_q_*_1_ and *E_q_*_2_ are the activation energies, eV; and *k* is the Boltzmann constant, eV/K. Table 2 presents the parameters of the approximation of the experimental data using the expression (1). Table 2 compares the results for HfO_2_ nanocrystals with a monoclinic structure, produced by nonaqueous sol–gel synthesis, with the findings of those annealed at 700 °C [49]. The present paper describes the temperature quenching processes for radioluminescence in the 2.5 eV band over a range of 160–300 K, using the Mott relation. In addition, the activation energy *E_q_*_2_ = 141 meV calculated in the current work agrees well with the estimate of 120 meV [49]. It should be emphasized that, at temperatures below 170 K, the nonradiative relaxation channel with *E_q_*_1_ = 15 meV dominates; see Figure 5. Previously, in [55], PL temperature quenching was investigated for cubic and monoclinic HfO_2_ nanoparticles obtained by sol–gel method. For the monoclinic sample, the authors also observed an increase in PL intensity as the temperature decreased from 300 to 200 K, which is consistent with our data. However, obtained experimental dependence was analyzed using a different relation, which does not allow us to compare the activation energy estimates.

The papers [48,56] theoretically estimated the trap depth for monoclinic HfO_2_. It was noted that negatively charged oxygen vacancies $V_{O}^{-}$ and $V_{O}^{2-}$ create occupied states located ~100 meV below the bottom of the conduction band. This magnitude is well consistent with the energies of thermal activation barriers determined in our experiments. Thus, thermally induced transitions of electrons from shallow traps based on $V_{O}^{-}$ and $V_{O}^{2-}$ vacancies to the conduction band may give rise to the temperature quenching of photoluminescence in the monoclinic NTs at hand. As for amorphous NTs, the configuration and charge state of an anion vacancy is more difficult to identify due to structural disorder [57]. Nevertheless, the characteristic values of the luminescence response and the temperature behavior of PL for amorphous and monoclinic nanotubes turn out to be almost equal; this may indicate similar parameters of short-range order in the local atomic surroundings of active emission centers. The above conclusion is quite compatible with the appearance of a monoclinic phase at fairly low temperatures in the hafnia samples with a variety of morphologies [4,58,59].

It is known that oxygen vacancies in HfO_2_ with monoclinic crystal structures are formed in positions with the coordination numbers of 3 (V_O3f_) and 4 (V_O4f_) [43,48,60]. Vacancies with a coordination number of 2 are additionally encountered in amorphous HfO_2_, but their proportion is negligibly small compared to V_O3f_ and V_O4f_ [61]. In the process, the formation energies of V_O3f_ and V_O4f_ centers are quite close [43]. As DFT-calculations argue, V_O4f_ vacancies are still more stable in monoclinic HfO_2_ [60]. In turn, amorphous HfO_2_ aggregates V_O3f_ defects twice as often as V_O4f_. The noticeably higher formation energy of the latter explains this circumstance (see calculations [61]).

In addition, according to [56], under conditions of excess hafnium, the formation energy of the V_O3f_ vacancies is lower and their prevalence may be due to, among other things, the synthesis method and the precursors used. In this work, the synthesis of the samples proceeds on the surface of hafnium foil during non-equilibrium electrochemical oxidation in a solution of ammonium fluoride and water in ethylene glycol. This means that it can be assumed that the growth of structures occurs under Hf-rich conditions. This allows one to assume that vacancies in the positions of three-coordinated oxygen are the primary basis for optically active centers responsible for the observable PL in the as-grown HfO_2_ nanotubes to emerge.

## 5. Conclusions

The current paper investigates the temperature effects in PL processes of hafnia nanotubes with amorphous and monoclinic structures. When excited by photons with an energy of 4.71 eV, the samples are found to exhibit luminescence spectra as a broad Gaussian band with a maximum of 2.30 ± 0.02 eV and 2.38 ± 0.02 eV for amorphous and monoclinic ones, respectively. The position and shape of the spectral bands remain unchanged over the entire temperature range of 7–296 K. During the first time we conducted the analysis of the PL temperature quenching processes in nanotubular hafnia, it revealed two channels of non-radiative relaxation of excitations with activation energies of 9 meV and 39 meV for amorphous nanotubes and 15 meV and 141 meV for monoclinic nanotubes. Anion vacancies $V_{O}^{-}$ and $V_{O}^{2-}$ appearing in the positions of three-coordinated oxygen could be the main contributors to the PL response and corresponding temperature quenching mechanisms in the synthesized hafnia nanotubes, according to the calculated values and a comparative analysis of independent data for different HfO_2_ nanostructures. The close characteristic values of the luminescence response and the temperature behavior of PL for amorphous and monoclinic nanotubes indicate similar parameters of short-range order in the local atomic surroundings of active emission centers.

## Figures and Tables

**Table 2:** Parameters of temperature quenching of the studied PL, calculated using the expression (1).

Sample	*S*_1_	*E_q_*_1_, meV	*S*_2_	*E_q_*_2_, meV	Source
Amorphous nanotubes	0.09 ± 0.05	9 ± 5	3.67 ± 0.45	39 ± 5	This work
Monoclinic nanotubes	1.97 ± 0.60	15 ± 1	919 ± 200	141 ± 30	This work
Monoclinic nanocrystals	—	—	400 ± 200	120 ± 10	[49]

## Data Availability

The original contributions presented in the study are included in the article/Supplementary Material, further inquiries can be directed to the corresponding author.

## Supplementary Materials

The following supporting information can be downloaded at: https://www.mdpi.com/article/10.3390/ma17225587/s1. Figure S1. The size distribution of nanotube inner diameters (a), outer diameters (b) and wall thickness (c). The size distribution of the outer diameters was previously shown in Shilov et al. [38]. Figure S2. XRD patterns of amorphous (top) and monoclinic (bottom) HfO_2_ nanotubes on anodized hafnium foil. The XRD data were measured in Shilov et al. [38]. A halo in the range of 20–38° indicates that the as-grown nanotubes have amorphous structure. Figure S3. Selected area electron diffraction of amorphous (left) and monoclinic (right) HfO_2_ nanotubes. Figure S4. The image obtained by applying the Fast Fourier Transform to Figure 1d.
