# Supplementary material for: Thermal Quenching of Intrinsic Photoluminescence in Amorphous and Monoclinic HfO2 Nanotubes
Source: Materials (Basel). 2024 Nov 15;17(22):5587. doi: 10.3390/ma17225587 (PMC11595964; doi:10.3390/ma17225587)
Supplement: Supplementary file 1 [file materials-17-05587-s001.zip › materials-3282222-supplementary.pdf]

Supplementary Materials

Thermal Quenching of Intrinsic Photoluminescence in Amorphous and Monoclinic HfO_2 Nanotubes

Artem Shilov [1], Sergey Savchenko [1], Alexander Vokhmintsev [1], Kanat Zhusupov [1,2] and Ilya Weinstein [1,3,*]

1 NANOTECH Centre, Ural Federal University, Mira Str., 19, 620002 Ekaterinburg, Russia; ao.shilov@urfu.ru (A.S.); s.s.savchenko@urfu.ru (S.S.); a.s.vokhmintsev@urfu.ru (A.V.); zhusupov.kanat@mail.ru (K.Z.)
2 Higher School of Metallurgy and Mining, Rudny Industrial University, 50 Let Oktyabrya Str., 38, Rudny 111500, Kazakhstan
3 Institute of Metallurgy, Ural Branch of the RAS, Amundsena Str., 101, 620108 Ekaterinburg, Russia

* Correspondence: i.a.weinstein@urfu.ru

The supplementary materials contain data on the characterization of nanotubular samples, such as size distributions (Figure S1), X-ray (Figure S2) and SAED patterns for amorphous and monoclinic hafnia nanotubes (Figure S3), and the image in Figure S4 obtained with using the Fast Fourier Transform of the TEM image shown in Figure 1d.

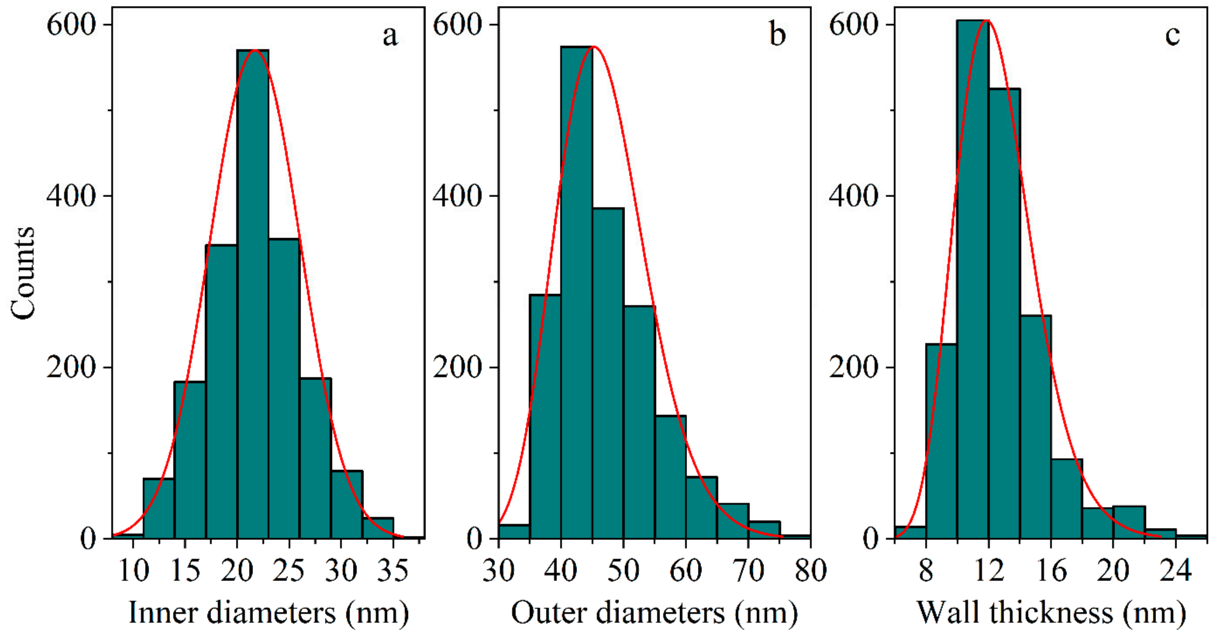

Figure S1 – The size distribution of nanotube inner diameters (a), outer diameters (b) and wall thickness (c). The size distribution of the outer diameters was previously given in Shilov et al. [38].

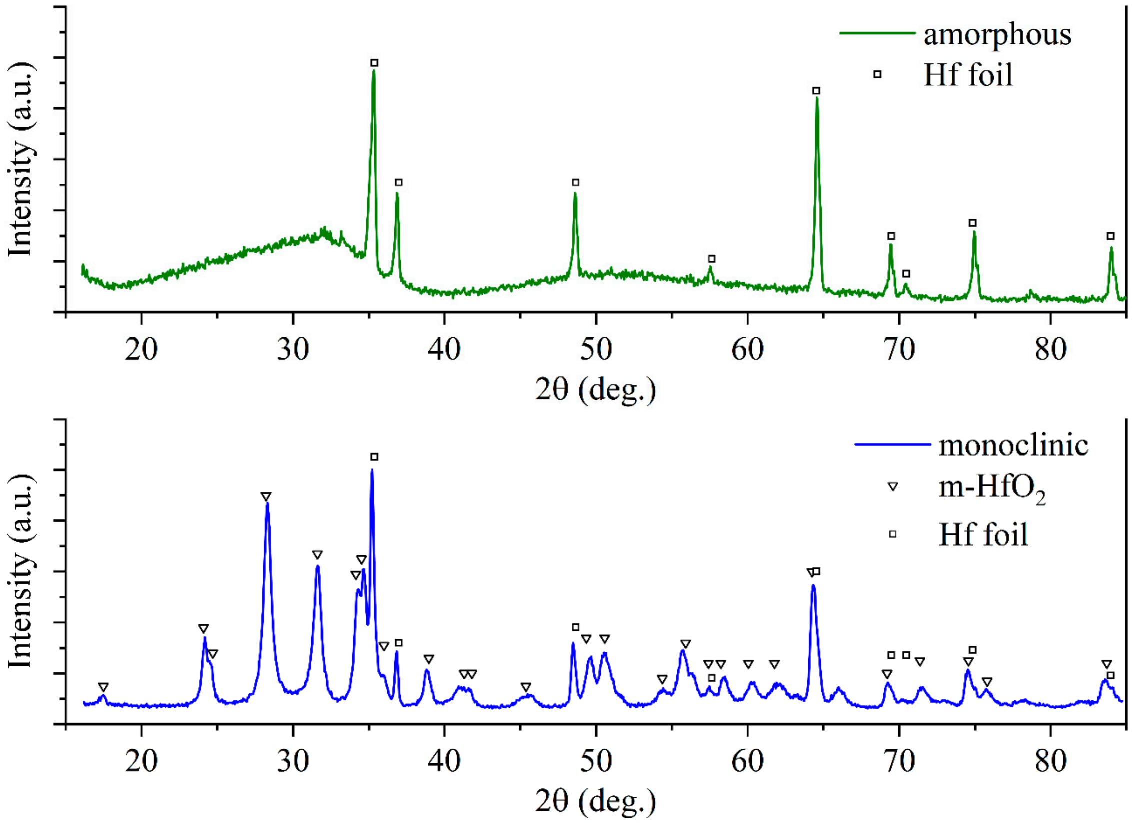

Figure S2 – XRD patterns of amorphous (top) and monoclinic (bottom) HfO_2 nanotubes on hafnium foil. The XRD data were measured in Shilov et al. [38]. A halo in the range of 20–38° indicates that the as-grown nanotubes have amorphous structure.

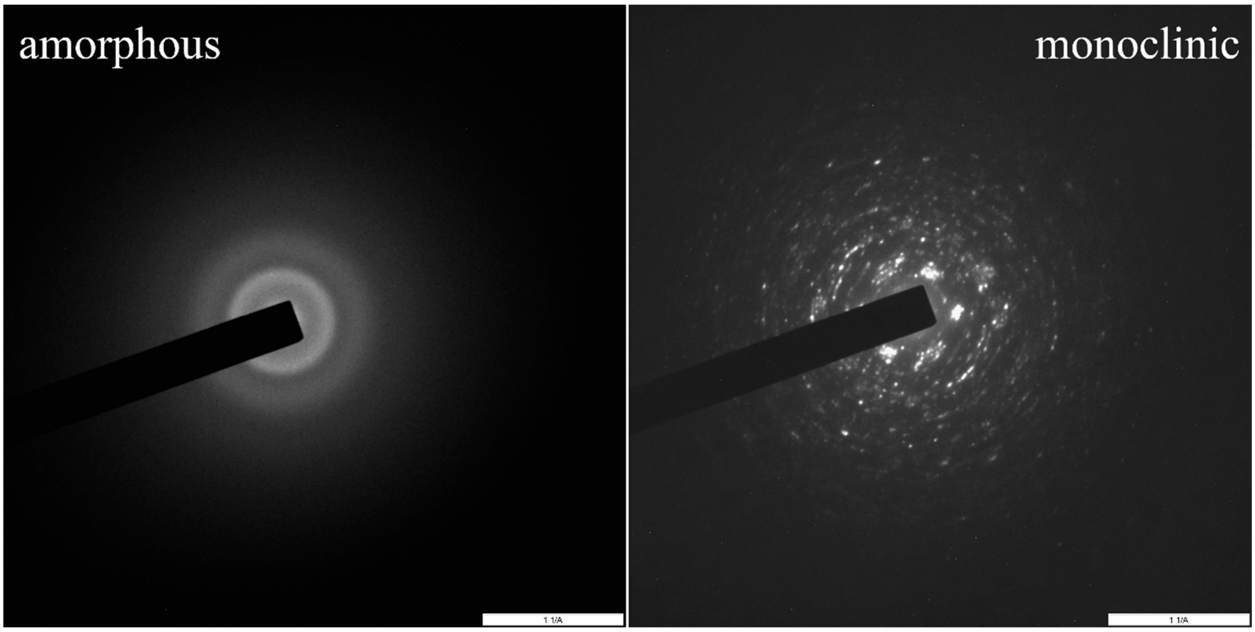

Figure S3 – Selected area electron diffraction of amorphous (left) and monoclinic (right) HfO_2 nanotubes.

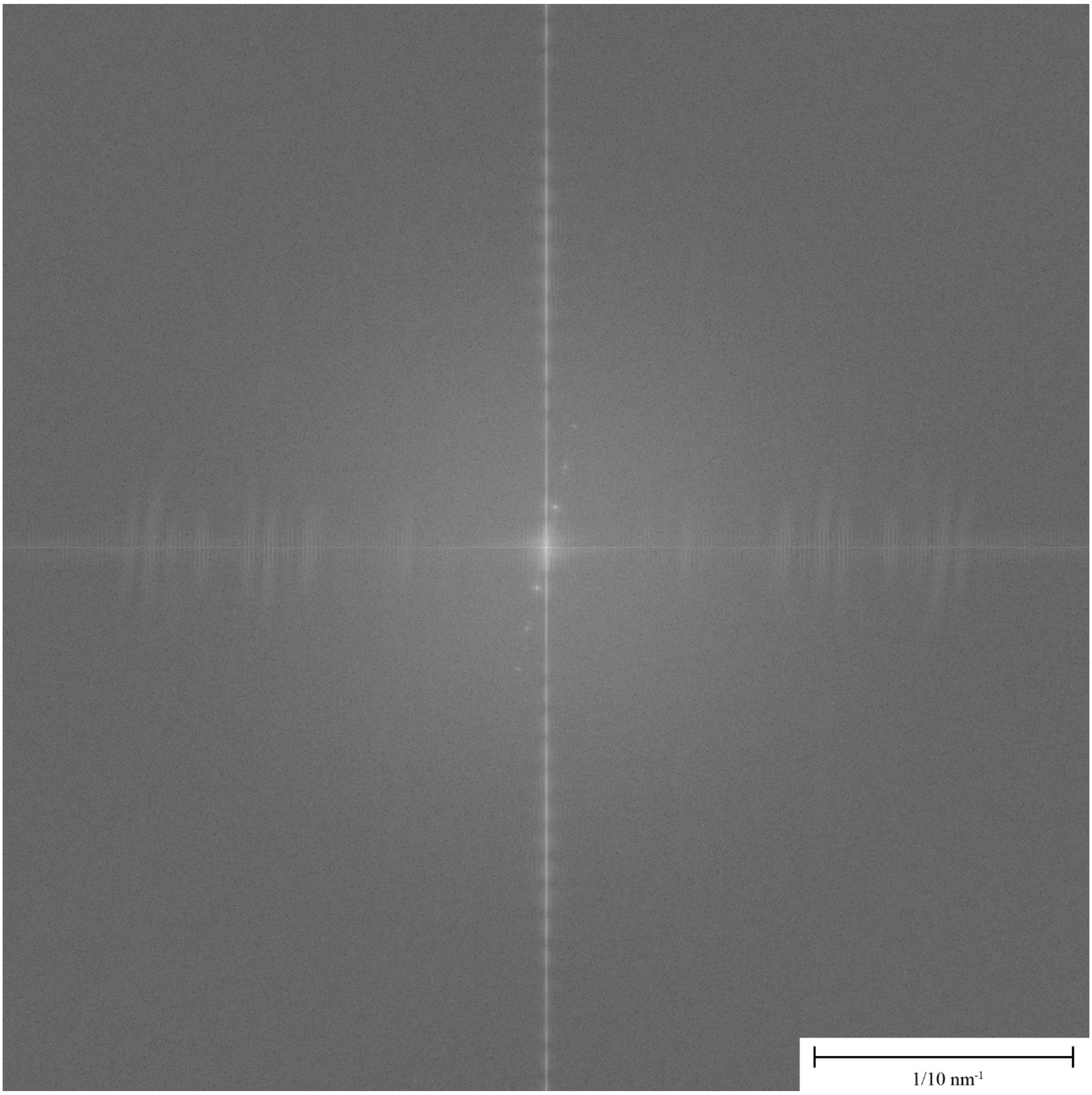

Figure S4 – The image obtained by applying the Fast Fourier Transform to Figure 1d.
